# A novel fusion circular RNA F-circBA1 derived from the *BCR-ABL* fusion gene displayed an oncogenic role in chronic myeloid leukemia cells

**DOI:** 10.1080/21655979.2021.1957749

**Published:** 2021-08-04

**Authors:** Yuan Tan, Zhenglan Huang, Xin Wang, Hongdan Dai, Guoyun Jiang, Wenli Feng

**Affiliations:** a Department of Clinical Hematology, School of Laboratory Medicine, Chongqing Medical University, Chongqing, China; b Department of Hematology, The First Affiliated Hospital of Chongqing Medical University, Chongqing, China

**Keywords:** bcr-abl, chronic myeloid leukemia, fusion circRNAs, proliferation

## Abstract

The *BCR-ABL* fusion gene plays a crucial role in the leukemogenesis of chronic myeloid leukemia (CML). The BCR-ABL oncoprotein encoded by this fusion gene has been extensively studied. However, research on whether *BCR-ABL* also affects circular RNAs (circRNAs) is limited. This study aimed to explore the new fusion circRNAs produced by *BCR-ABL* and their role in CML cells. In this study, we identified a novel fusion circRNA, named F-circBA1, originating from *BCR-ABL* in K562 and K562/G01 cells using quantitative reverse transcriptase-polymerase chain reaction (qRT-PCR) and Sanger sequencing. qRT-PCR of the nuclear RNA and cytoplasmic RNA were separated, indicating that F-circBA1 was mainly localized in the cytoplasm. Cell counting kit-8 assay and flow cytometry showed that F-circBA1 knockdown by shRNA prevented the proliferation of K562 and K562/G01 cells, and the cell cycle was arrested at G2/M. Mechanically, dual-luciferase reporter assay and western blotting assay showed that F-circBA1 sponged miR-148-3p and F-circBA1 silencing decreased CDC25B expression in vitro. Furthermore, the results of the murine leukemogenesis model showed that F-circBA1 knockdown suppressed leukemogenesis in vivo. Besides, we found the existence of F-circBA1 in some patients with *BCR-ABL*-positive CML. In conclusion, these results demonstrate the presence of F-circBA1 and its oncogenic role in CML cells.

## Introduction

Chronic myeloid leukemia (CML) is a malignant myeloproliferative disorder derived from hematopoietic stem cells [1]. More than 95% of patients with CML are characterized by the reciprocal chromosome translocation t (9;22) (q34; q11) [2]. The BCR-ABL oncoprotein encoded by the *BCR-ABL* fusion gene is currently considered as the main cause of CML [3]. Although the tyrosine kinase inhibitor imatinib is effective in CML treatment, approximately 25% of patients suffer from emerging resistance or intolerance [4]. Therefore, it is necessary to further explore the molecular mechanisms of CML to find more effective therapeutic strategies to overcome these problems.

Circular RNAs (circRNAs) are a type of single-stranded covalently closed non-coding RNAs that play important roles in various biological processes [5,6]. CircRNAs are more stable compared with linear RNAs due to their special circular structure, which can effectively resist degradation induced by exonucleases [7]. Besides, increasing evidence demonstrates that that circRNA is also involved in the pathogenesis of cancer, including hematologic malignancies [8]. CircRNAs can serve as microRNA sponges to regulate specific cellular pathways or directly interact with proteins to regulate their function [9].

Recently, emerging evidence has demonstrated that cancer-associated chromosomal translocations does not only generate oncoprotein but also produces fusion circRNAs (F-circRNAs) associated with leukemogenesis [10]. These F-circRNAs can promote the proliferation of leukemia cells, and synergistic fusion proteins leading to the occurrence of leukemia. Fusion circRNAs encoded by fusion genes are also been reportedly involved in the progression of non-small cell lung cancer [11]. Given the important role of *BCR-ABL* in the pathogenesis of CML, it is of great value to investigate whether *BCR-ABL* encodes F-circRNAs associated with leukemogenesis. Currently, two F-circRNAs encoded by *BCR-ABL* have been reported. One is circBA9.3 [12], which can efficiently promote the proliferation and inhibit apoptosis of CML cells. The other one, named circBA1 [13], can inhibit cell proliferation in CML. However, they have not yet been validated by in vivo models. It is also unclear whether there are new F-circRNAs produced by *BCR-ABL*.

In this study, we hypothesized that there are other potential *BCR-ABL*-derived F-circRNAs in CML cells, and our main goal was to identify new *BCR-ABL*-derived fusion circRNAs and explore their roles in CML cells and mouse models. Here, we identified a new F-circRNA produced by *BCR-ABL* in CML cell lines (K562 and K562/G01) and named it F-circBA1. We confirmed the proliferation promoting effect of F-circBA1 in vivo and in vitro. In addition, we demonstrated the presence of F-circBA1 in some CML patient samples. This finding suggests that *BCR-ABL* can encode more than one kind of circRNA and different circRNAs encoded by *BCR-ABL* may play different roles in CML.

## Materials and methods

### Cell culture

Human leukemia cell lines K562 (immortalized cell lines derived from human chronic myeloid leukemia samples), K562/G01 (imatinib-resistant cell lines screened by long-term low-dose imatinib treatment), THP1 (human acute monocytic leukemia cell line), HL-60 (neutrophilic human acute promyelocytic leukemia cell line), and NB4 (human acute promyelocytic leukemia cell line) [14] were cultured in RPMI 1640 (Gibco, USA) containing 10% fetal bovine serum (FBS; Gibco, USA). Human embryonic kidney cells HEK-293 T were maintained in DMEM (Gibco, USA) supplemented with 10% FBS. All cell lines were maintained at 37°C in a 5% CO_2_ incubator.

### Patient samples

Bone marrow (BM) of nine patients with CML were supplied by the second affiliated hospital of Chongqing Medical University (Chongqing, China). Mononuclear cells were collected via Ficoll gradient purification (Tbd science, Tianjin, China). Details of the clinical characteristics of patients with CML are provided in Table S1. Informed consent was obtained for experimentation with human samples.

### RNA extraction, RNase R treatment, and PCR

Total RNA was isolated using TRIZOL (Invitrogen). The nuclear RNA and cytoplasmic RNA were separated using RNA isolation kits (Thermo Fisher, AM1921). For RNase R treatment, total RNA (5 μg) was incubated 15 min at 37°C with RNase R (Geneseed, Guangzhou, China) 2 U/μg and then 70°C for 10 min to inactivate the enzyme activity. RNA was directly reverse transcribed to cDNA with PrimeScript™RT reagent kit (Takara, Japan) using random primers. CircRNA encoded by *BCR-ABL* was analyzed using PCR as reported previously [10]. PCR reactions were performed using Premix Taq™ (Takara, Japan) with BIO-RAD T100TMThermal Cycler PCR system. Cycling conditions were as follows: 3 min at 94°C for the initial denaturation; 40 cycles of 30 s at 94°C, 30 s at 55°C, and 30 s at 72°C; final extension for 10 min at 72°C. The melting curve was 65 ~ 95°C at the rate of 0.5°C/0.05 s [15]. PCR products were observed using 2% agarose gel electrophoresis. QIAquick extraction kit (QIAGEN) was used to purify and recycle PCR products from the gel for Sanger sequencing. Quantitative real-time PCR (qRT-PCR) was performed using TB Green Premix Ex Taq II (Takara, Japan). Primer sequences are provided in Table S2.

### Lentivirus-mediated cell infection

The short hairpin (shRNA) targeting the backsplice junction of F-circBA1 and the non-targeting shRNA were purchased from Genechem (Shanghai, China). The target sequences for the backsplice junction of F-circBA1 shRNA were 5’- CTACATCACGCCAGACTGT −3’ and 5’- CATCACGCCAGACTGTCCA −3’. K562 and K562/G01 cells were infected with shRNA lentivirus targeting F-circBA1 for 72 h in the presence of 5 μg/mL polybrene (Sigma, USA), following 4 μg/mL puromycin (Sigma, USA) selection until the blank control group died. qRT-PCR was used to screen K562 and K562/G01 cells with stably silenced F-circBA1 for subsequent experiments.

### Cell counting kit-8 (CCK-8) and colony formation assay

CCK-8 and colony formation assays were performed as previously described [14]. Stable F-circBA1-knockdown cell lines and control cell lines were seeded at 3000 cells per well in 96-well plates with 100 μl RPMI 1640 and 10% FBS, and cultured at 37°C in a 5% CO_2_ incubator. Exactly 10 μL of CCK-8 solution was added into each well at the indicated time point and incubated for another 3 h at 37°C. Cell viability was detected by measuring the absorbance at 450 nm using a microplate reader (Eon, Bio Teck, USA).

### Apoptosis and cell cycle analysis

K562 and K562/G01 cells were collected 72 h after lentivirus infection. For the apoptosis assay, 1 × 10^6^ cells were washed with cold phosphate buffered saline (PBS) and resuspended in 1 ml of 1× binding buffer. Then, the cells were stained with 5 μl of phycoerythrin (PE) and 5 μl of 7-aminoactinomycin D (7-AAD) successively using Annexin PE/7-AAD Apoptosis Detection Kit (Vazyme, Nanjing, China) according to the manufacturer’s instructions. For cell cycle assay, 1 × 10^6^ cells were washed and resuspended in cold PBS and incubated in ice-cold 70% ethanol for 4 h. Coulter FC500 flow cytometry (Beckman, CA, USA) was applied to detect apoptosis and cell cycle [16].

### RNA immunoprecipitation assay (RIP)

RIP experiments were performed using the BersinBio^TM^ RIP kit (BersinBio, Guangzhou, China) following the manufacturer’s instructions. Approximately 2 × 10^7^ cells were collected and resuspended in 800 μL of RNA lysis buffer and then incubated with 5 μg of anti-AGO2 peptide (ab32381, Abcam, USA) or anti-IgG antibody rotating at 4°C for 16 h. Exactly 20 µL of protein A/G beads was added to each sample and incubated with rotation at 4°C for 2 h. Next, the mixtures were treated with proteinase K buffer. Finally, the immunoprecipitated RNAs were extracted via phenol-chloroform purification and detected using qRT-PCR.

### Dual-luciferase activity assay

MiR-148b-3p mimics were synthesized by Biomics (Nantong, China). The inclusion of the F-circBA1 sequence containing an miR-148b-3p binding site was cloned into the pmirGLO dual-luciferase reporter (Genecreate, Wuhan, China) to construct wild-type reporter F-circBA1-wt. The mutant reporters F-circBA1-mut were built by mutating seven nucleotides, which were complementary to miR-148b-3p on F-circBA1. 293 T cells were seeded in 96-well plates, and then co-transfected using Lipofectamine 2000 (Invitrogen, Thermo Fisher Scientifc, Inc.) at approximately 60% confluence with wild-type or mutant F-circBA1 reporter plasmids, and miR-148b-3p mimics or miR-NC. After transfection for 48 h, luciferase activity was measured using the Dual-Luciferase Reporter Assay Kit (Promega). The relative luciferase activity was determined by calculating the ratio of firefly fluorescence to Renilla fluorescence.

### Western blotting

Western blotting analysis was performed as previously described [14]. Cells were lysed using the RIPA buffer (Beyotime, China). The protein concentration was determined using bicinchoninic acid (BCA) protein assay kit (Beyotime). Proteins were separated via SDS-PAGE and transferred onto PVDF membranes (Millipore, Boston, MA, USA). Membranes were detected using enhanced chemiluminescence substrate (ECL) (Millipore, USA). The images were acquired using the Bio-Rad Gel Imaging System on cool image workstation II (Viagene, USA). Primary antibodies were as follows: Polyclonal Goat anti-Cdc25B (AF1649, 1:6000, R&D, CA, USA); rabbit anti-β-actin (3700, 1:5000, CST); goat anti-rabbit (ZB2301, 1:5000, USA); rabbit anti-goat (ZB2306, 1:5000, USA).

### Immunofluorescence assay

Cells were collected and washed three times with ice-cold PBS and then fixed using 4% paraformaldehyde. Then, the cells were permeabilized using 0.2% Triton X-100 for 15 min at 37°C, blocked with 5% goat serum for 2 h and incubated with the primary antibody (1:200 in 5% goat serum) overnight at 4°C. Next, the cells were incubated with a fluorescently-labeled secondary antibody (Introvigen, USA) for 1 h in the dark at 37°C and stained with diluted DAPI (1:1000 in PBS). Finally, the cells were observed using a Nikon A1R microscope under a 40x oil immersion objective (Numerical aperture, 1.4).

### Murine leukemogenesis model

Five- to six-week-old female NOD/SCID mice were specially reared without pathogens in the Experimental Animal Center of Chongqing Medical University. Mice received sub-lethal radiation of 250 cGy before injection. After 2–3 h, mice were injected through the tail vein with 5 × 10^6^ shRNA-transformed K562/G01 cells in 200 μL PBS. The control group (PBS) received sub-lethal radiation of 250 cGy and then injected with 200 μl of cell-free PBS. The body weight, white blood cell count (WBC) and mental state of mice were monitored weekly. For WBC count, 10 μl of peripheral blood was collected and dissolved in 190 μl of 3% glacial acetic acid using the tail cutting method. After standing on ice for 10 min, peripheral blood WBC count was determined [17]. BM and peripheral blood from xenograft mice were treated with erythrocyte lysis buffer (Biolegend, USA). The GFP^+^ % of transduced K562/G01 cells was calculated using Coulter FC500 flow cytometer (Beckman, CA, USA) and analyzed using the FlowJo software. All animal experiments were conducted following the Ethical Guidelines for Animal Experiment Institutions and approved by the Ethics Committee of Chongqing Medical University.

### Hematoxylin & eosin (H&E) and Wright’s staining

For Wright’s staining, BM cells were collected after the mice died and washed twice with precooled PBS. Then, cells were dissolved in 20–50 μl of PBS solution and smeared. After the glass slides dried naturally they were dipped into Wright’s dye solution A for 30 s, then into Wright’s dye solution B (1.5× the previous volume) for 1 min. After evenly dying the slides, they were gently rinsed under running water for 1 min. After the glass slides were dried, a microscopic examination was carried out and images were acquired [18]. For H&E staining, the liver and spleen tissues of mice were stored in 4% paraformaldehyde after the mice died and staining was performed.

### Statistical analysis

The unpaired t-test was used for comparison between two groups, and one-way ANOVA followed by Dunnett’s or Tukey’s test for comparison between multiple groups. Data were expressed as the mean ± standard error (SEM) or standard deviation (SD) of three independent experiments. Statistical analysis was performed using GraphPad Prism software version 7 (GraphPad Software, Inc.). Different levels of statistical significance were denoted by P-values (*P < 0.05, **P < 0.01, ***P < 0.001).

## Results

In this study, we hypothesized that *BCR-ABL* may encode for circRNAs other than the ones identified so far playing different roles in CML. Indeed, a novel F-circRNA was discovered here using CML cell lines (K562 and K562/G01) via PCR and sequencing techniques, named F-circBA1.We demonstrated the pro-proliferation effect of F-circBA1 in vivo and in vitro using the CCK-8 assay, flow cytometry, and a murine leukemogenesis model.

### Identification and characterization of F-circBA1 in CML cells

**Figure 1.**
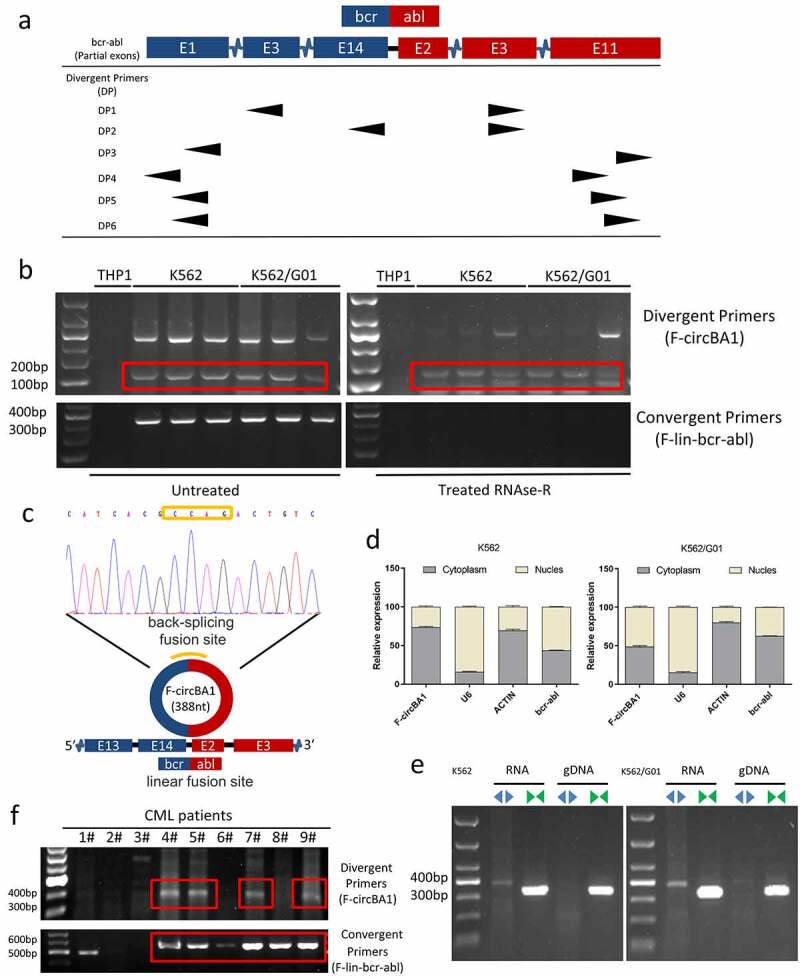
Identification and characterization of F-circBA1 in chronic myeloid leukemia. (a) Schematic representation of the position of the six divergent primer pairs on *BCR-ABL*. (b) PCR analysis of RNAs in leukemic cells harboring a *BCR-ABL* translocation (K562 and K562/G01) or other translocations (THP-1) for the identification of F-circBA1. Divergent primers were used to scout F-circBA1 and convergent primers are used to detect bcr-abl. RNAse R treatments were used to detect the stability of the product. (c) The sequence and reverse splicing site of F-circBA1 were verified via Sanger sequencing. (d) The expression levels of F-circBA1, *BCR-ABL* mRNA, U6 (Nuclear Control), and ACTIN (Cytoplasm Control) in the cytoplasm and nucleus of K562 and K562/G01 cells were analyzed using qRT-PCR. (e) F-circBA1 and *BCR-ABL* were amplified via PCR at RNA and gDNA levels with divergent and convergent primers, respectively. (f) The presence of F-circBA1 in patients with CML was detected via PCR

To explore whether the F-circRNAs were derived from *BCR-ABL*, multiple divergent primer pairs were designed on both linear fusion sites on either end of *BCR-ABL* (Figure 1a). Divergent primers were used to amplify possible F-circRNAs originated from *BCR-ABL*. The results of agarose gel electrophoresis showed that most of the divergent primers could detect the positive products (Supplementary Figure 1a-f). However, we found that only one product contained both *BCR* and *ABL* regions with the backsplice junction between the 5′ head of *BCR* exon 13 and the 3′ tail of *ABL* exon 3 (Figure 1b). We obtained a complete sequence of the fusion circRNA with 388 nucleobases and named it F-circBA1 (Figure 1c). F-circBA1 contained complete 14 exons of *BCR* and exon 2 of *ABL*, along with a partial sequence of BCR exon 13 and ABL exon 3. More specifically, the complete sequence of F-circBA1 was not retrieved from the NCBI and circBase databases. Besides, we did not detect the presence of F-circBA1 in *BCR-ABL*-negative leukemia cells THP-1, HL-60, and NB4 (Supplementary Figure S2).

We next characterized F-circBA1 by investigating its physical properties. Total RNA was treated with RNase R and then subjected to PCR with divergent and convergent primers. Compared with the linear counterpart *BCR-ABL*, F-circBA1 was relatively resistant to RNase R digestion (Figure 1b). Then, the intracellular localization of F-circBA1 was identified via qRT-PCR, detecting the nuclear and cytoplasmic RNA. *U6* was the nuclear internal reference, *ACTIN* was the cytoplasmic internal reference, and F-circBA1 was distributed in both cytoplasm and nucleus. The results showed that in K562 cells, F-circbA1 was preferentially localized in the cytoplasm, and in K562/G01 cells, F-circbA1 was distributed in both cytoplasm and nucleus (Figure 1d). PCR results also demonstrated that F-circBA1 was derived from RNA rather than gDNA (Figure 1e), which confirmed the formation of circular transcripts. These data demonstrated the presence of a new circRNA F-circBA1 produced by the *BCR-ABL* fusion gene.

### F-circBA1 was detected in patients with CML

To investigate whether F-circBA1 exists in the clinic, we measured F-circBA1 expression in samples from patients with CML. However, the existence of F-circBA1 was only detectable in 9/14 patients and not all *BCR-ABL*-positive patients with CML (Figure 1f and Supplementary Figure S3), which might be due to unknown causes such as low enrichment of F-circBA1 or differences in the in vivo microenvironment. These results suggested that the presence or absence of F-circBA1 in vivo might be related to individual differences, and the specific reasons are worth further investigation.

### F-circBA1 silencing of inhibits K562 and K562/G01 cell proliferation

**Figure 2.**
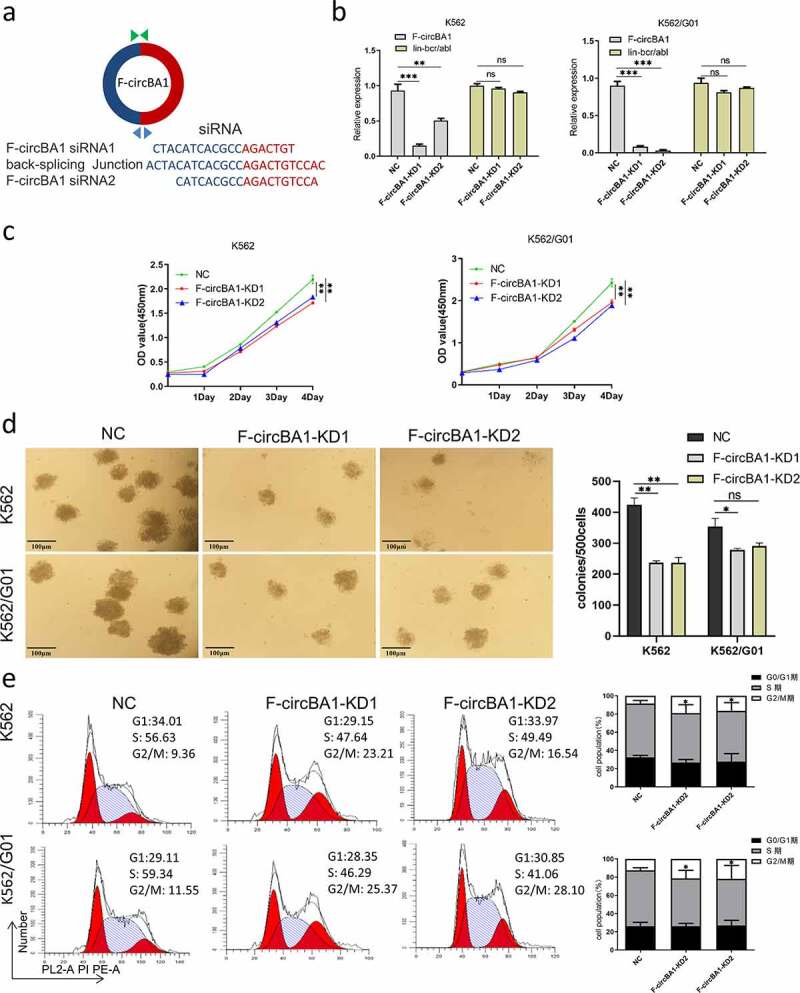
F-circBA1 plays an oncogenic role in CML cells in vitro. (a) Schematic representation of the junction site of F-circBA1 and two targeted siRNAs. (b) The expression levels of F-circBA1 and *BCR-ABL* in CML cells after lentivirus infection were analyzed using qRT-PCR. (c, d) The proliferation of CML cells after F-circBA1 knockdown was detected using CCK-8 and colony formation assays. (e) The cell cycle of *BCR-ABL* leukemia cells after F-circBA1 knockdown was detected using flow cytometry. *P < 0.05, **P < 0.01, ***P < 0.001

Since *BCR-ABL* is the main cause of CML pathogenesis, we asked whether F-circBA1, as one of the transcription products of *BCR-ABL*, plays a functional role in CML. Two shRNAs were designed against the F-circBA1 backsplice junction site with a scrambled siRNA sequence as the control (Figure 2a). In K562 and K562/G01 cells infected with a lentivirus encoding shRNA1 or shRNA2, the level of F-circBA1 was significantly decreased, while the expression level of the homologous *BCR-ABL* mRNA was not affected (Figure 2b). Next, we detected the effect of F-circBA1 on the proliferation of CML cells. CCK-8 assay results showed that downregulation of F-circBA1 reduced the proliferation of K562 and K562/G01 cells (Figure 2c). Colony formation assay showed that F-circBA1 silencing inhibited the colony-forming ability of K562 and K562/G01 cells (Figure 2d). Furthermore, we investigated whether F-circBA1 knockdown could affect apoptosis and the cell cycle. The results showed that F-circBA1 knockdown had a non-significant effect on cell apoptosis (Supplementary Figure S4); however, induced cell cycle arrest at the G2/M phase (Figure 2e). These results indicated that F-circBA1 silencing inhibited CML progression.

### F-circBA1 promotes leukemogenesis in vivo

**Figure 3.**
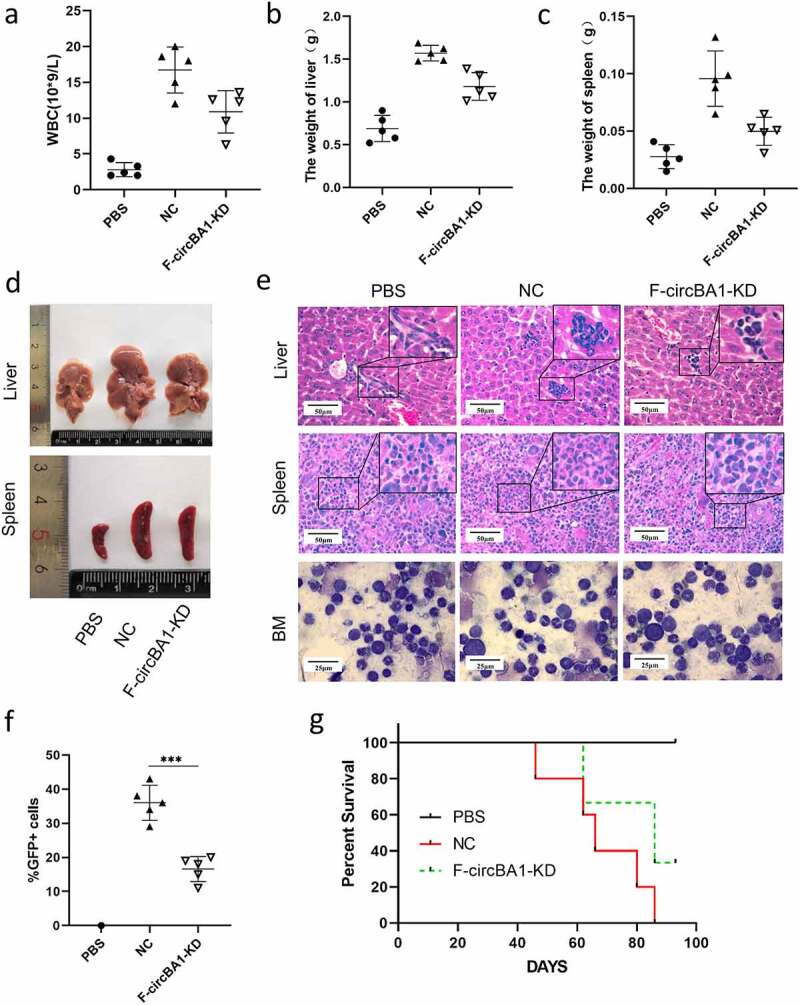
F-circBA1 promotes leukemogenesis in vivo. (a) Total white blood cell count of peripheral in PBS, NC, and F-circBA1-KD groups were determined. (b-d) The weight and size of liver and spleen in PBS, NC, and F-circBA1-KD groups were determined. (e) The infiltration of CML cells in the indicated organs of each group was detected via histopathological and bone marrow cytological analysis. (f) The percentage of GFP^+^ cells in the peripheral blood of each group were tested using flow cytometry. (g) Survival curves were analyzed using Kaplan-Meier methods. *P < 0.05, **P < 0.01, ***P < 0.001

To further investigate the effects of F-circBA1 on leukemogenesis in vivo, the same number of K562/G01 cells stably expressing F-circBA1 shRNA (F-circBA1-KD) or control shRNA (NC) were intravenously injected into NOD-SCID mice through the tail vein. The control group was injected with 200 μl of cell-free PBS. We found that the WBC counts of mice that received F-circBA1-silenced K562/G01 cells were significantly lower than those in mice that received K562/G01 cells expressing shNC (Figure 3a). The results showed that the F-circBA1-KD group developed smaller spleens and livers compared with the NC group (Figure 3b–d). Furthermore, results of H&E and Wright’s staining suggested that the number of K562/G01 cells in the BM, spleen, and liver of the F-circBA1-KD group was lower than that in the NC group, indicating that F-circBA1 could regulate the infiltration of BCR-ABL leukemia (Figure 3e). Immunofluorescence assay also revealed that BCR-ABL expression was decreased in the liver, spleen, and BM in the F-circBA1-KD group compared with that the NC group (Supplementary Figure 5). Flow cytometry analysis exhibited that the percentage of GFP^+^ cells in the peripheral blood of the mice in the F-circBA1-KD group was lower than that in the NC group (Figure 3f), suggesting that F-circBA1-KD inhibited the proliferation of K562/G01 in mice. The survival time of mice in the F-circBA1-KD group was significantly extended compared with that of the NC group (Figure 3g). In summary, these results suggested that F-circBA1 silencing slowed the progression of CML in vivo.

### F-circBA1 acts as an miR-148b-3p sponge

**Figure 4.**
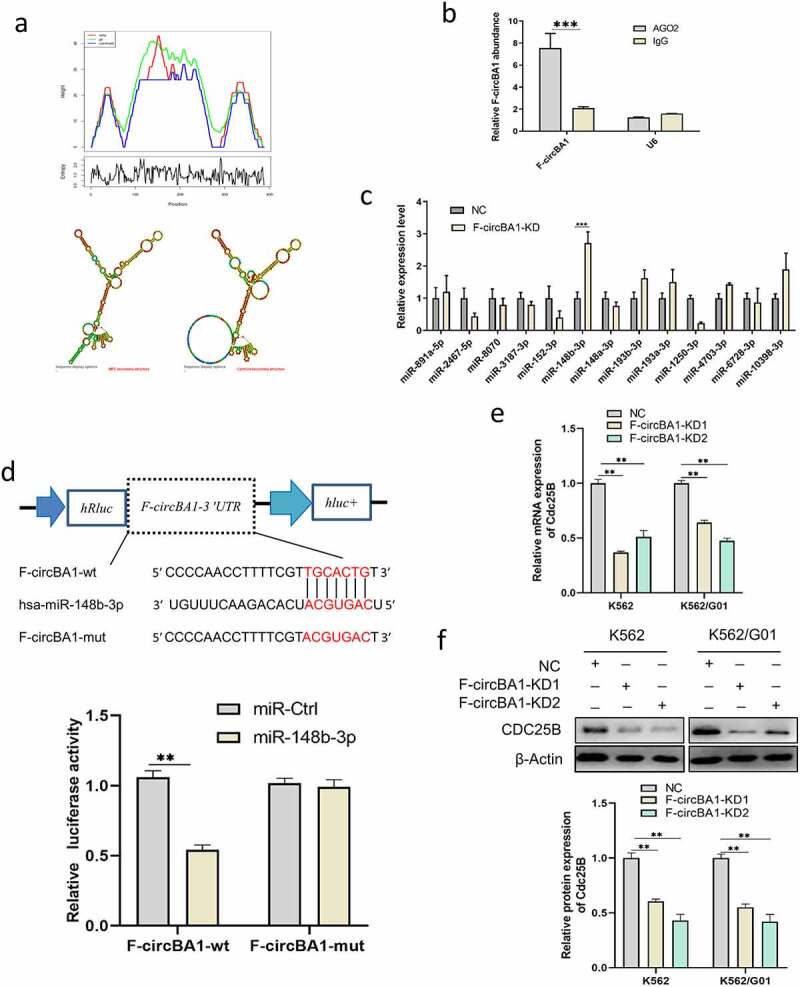
F-circBA1 acts as an miR-148b-3p sponge and has clinical significance. (a) Schematic representation of the secondary structure of F-cirBA1. (b) The binding ability of F-circBA1 and AGO2 was detected via AGO2 RIP and qRT-PCR analysis. (c) The expression level of the target miRNA of F-circBA1 after F-circBA1 knockdown in CML cells was detected via qRT-PCR. (d) The binding ability of F-circBA1 and miR-148b-3p was determined via luciferase assay. (e, f) The mRNA and protein expression levels of CDC25B in CML cells after F-circBA1 knockdown were detected using qRT-PCR and WB. *P < 0.05, **P < 0.01, ***P < 0.001

Given that F-circBA1 was predominantly localized in the cytoplasm, we asked whether F-circBA1 could sponge an miRNA to regulate their target genes. First, we predicted the secondary structure of F-circBA1 using MFOLD [19], and the results revealed the circular structure of F-circBA1 and the possibility of miRNA binding (Figure 4a). Next, we used the CircInteractome Database to predict the potential target miRNAs and selected 13 candidate miRNAs for further validation (Table S3). We performed qRT-PCR analysis with RIP of *AGO2*, and the results showed that F-circBA1 could bind to AGO2 (Figure 4b) [20]. qRT-PCR results suggested that miR-148b-3p was significantly enhanced in the F-circBA1-KD group compared with other candidates (Figure 4c). Dual-luciferase activity demonstrated that miR-148b-3p mimics decreased the luciferase activity of the F-circBA1-wt, but not the F-circBA1-mut (Figure 4d), indicating that F-circBA1 could bind to miR-148b-3p directly.

To investigate whether F-circBA1 plays an oncogenic role by affecting the expression of the miR-148b-3p target genes, we predicted the potential target genes of miR-148b-3p using picTar, miRmap, miRanda, PITA, miRTarBase, and TargetScan. qRT-PCR and western blotting results showed that F-circBA1 knockdown reduced CDC25B [21,22] expression in K562 and K562/G01 cells at both mRNA and protein levels (Figure 4e and f). This data suggested that the G2/M phase cell cycle arrest caused by F-circBA1 knockdown might be due to the release of CDC25B degradation by miR-148b-3p.

## Discussion

Cancer-associated chromosomal translocations that not only generate an oncoprotein but also produce F-circRNAs associated with leukemogenesis have been identified. However, there are limited studies on the role of F-circRNAs in CML, and these limited studies did not involve in vivo model validation. Given the current treatment bottleneck of CML, it is necessary to further explore the molecular mechanism. Considering that the *BCR-ABL* fusion gene plays an important role in CML, we investigated its impact on circRNAs. In our study, we found a novel F-circRNA (F-circBA1) encoded by *BCR-ABL*, which promoted the proliferation of *BCR-ABL*-positive CML cell lines.

CircRNAs generated by fusion genes support the survival of leukemic cells or inhibits cell proliferation [10,23]. These studies demonstrated that the fusion oncogenic proteins encoded by cancer-related chromosomal translocations are not the only factors related to the pathogenesis of the disease and unraveled new possible opportunities for therapeutic intervention. However, current research on circRNAs is mainly focused on circRNAs produced by non-fusion genes and the effects of these circRNAs on solid tumors. For example, Han et al. found a novel circular RNA in the lipopolysaccharide-induced acute lung injury model of MRC-5 cells [24]. In addition, circ_0000467 has been reported to regulate the development of colorectal cancer via the miR-382-5p/EN2 axis [25]. The present study was aimed at finding the fusion circRNAs produced by fusion genes in CML and their effects on the progression of CML in vitro and in vivo. At present, whether the *BCR-ABL* fusion gene could also affect the circRNAs has been poorly explored. Two fusion circRNAs originated from *BCR-ABL* have been reported [12,13], but they exhibit opposite effects on CML cells. Our study revealed another novel F-circRNA (F-circBA1) which might play an oncogenic role in CML. As one of the transcriptional accessories of *BCR-ABL*, F-circBA1 contained fusion sites of linear *BCR-ABL* between BCR exon 14 and ABL exon 2. As a product of back-splicing, F-circBA1 contained a new junction site between BCR exon 13 and ABL exon 3. Our findings suggested that *BCR-ABL* could encode for more than one kind of circRNA and extended the conclusion of observational studies.

In the present study, we found that F-circBA1 silencing inhibited the progression of CML by inducing cell cycle arrest at G2/M and had a non-significant effect on apoptosis. Previous studies have demonstrated that circRNAs can act as a sponge for miRNAs to regulate the expression of target genes in a variety of human diseases, including leukemia. For example, circRBPMS inhibits bladder cancer progression by regulating RAI2 expression through sponging miR-330-3p [26]. In the present study, F-circBA1 sponged miR-148b-3p, thereby releasing its inhibition on CDC25B expression. CDC25B is an important regulator of cell cycle progression and is overexpressed in many cancer types, and it directly binds to miR-148b-3p [21,22]. When CDC25B is downregulated, the cell cycle is arrested at the G2/M phase [22]. Thus, F-circBA1 silencing could reduce CDC25B expression and induce cell cycle arrest at G2/M. Also, the results of in vivo leukemogenesis experiments demonstrated that F-circBA1 regulates the infiltration of CML. Collectively, our study indicated that F-circBA1 regulates the expression of CDC25B and affects the cell cycle of CML cells by sponging miR-148b-3p.

As for the clinical significance of F-circBA1, our study indicated that F-circBA1 existed in some patients with *BCR-ABL*-positive CML. However, the causes are still unsolved. Possible reasons could be low enrichment of F-circBA1 or differences in the microenvironment in vivo [27]. This is an interesting study area, and the specific reasons deserve further investigation. Future studies are necessary to explore the regulation of F-circBA1 production and differences among patients.

## Conclusion

In summary, we identified a novel fusion circRNA F-circBA1 encoded by *BCR-ABL*. F-circBA1 was mainly localized in the cytoplasm to arrest the cell cycle at the G2/M phase in K562 and K562/G01 cells. In addition, F-circBA1 knockdown could impair the leukemogenic ability of CML cells in vivo. However, the specific mechanism of F-circBA1 and its expression in clinical patients still need to be studied further. Our findings provide a new perspective for the study of the pathogenesis of CML and novel therapeutic targets.

## Supplementary Material

Supplemental Material

## Data Availability

The data used to support the findings of this study are available from the corresponding author upon request.

## Highlights

1. A novel fusion circRNA (F-circBA1) was identified in CML cell lines.
2. F-circBA1 is derived from the *BCR-ABL* fusion gene.
3. F-circBA1 displayed an oncogenic role in chronic myeloid leukemia cells.
4. F-circBA1 exists in some patients with *BCR-ABL*-positive CML.
