# Supplementary figures and images for: A novel fusion circular RNA F-circBA1 derived from the BCR-ABL fusion gene displayed an oncogenic role in chronic myeloid leukemia cells
Source: Bioengineered. 2021 Aug 4;12(1):4816–27. doi: 10.1080/21655979.2021.1957749 (PMC8806869; doi:10.1080/21655979.2021.1957749)

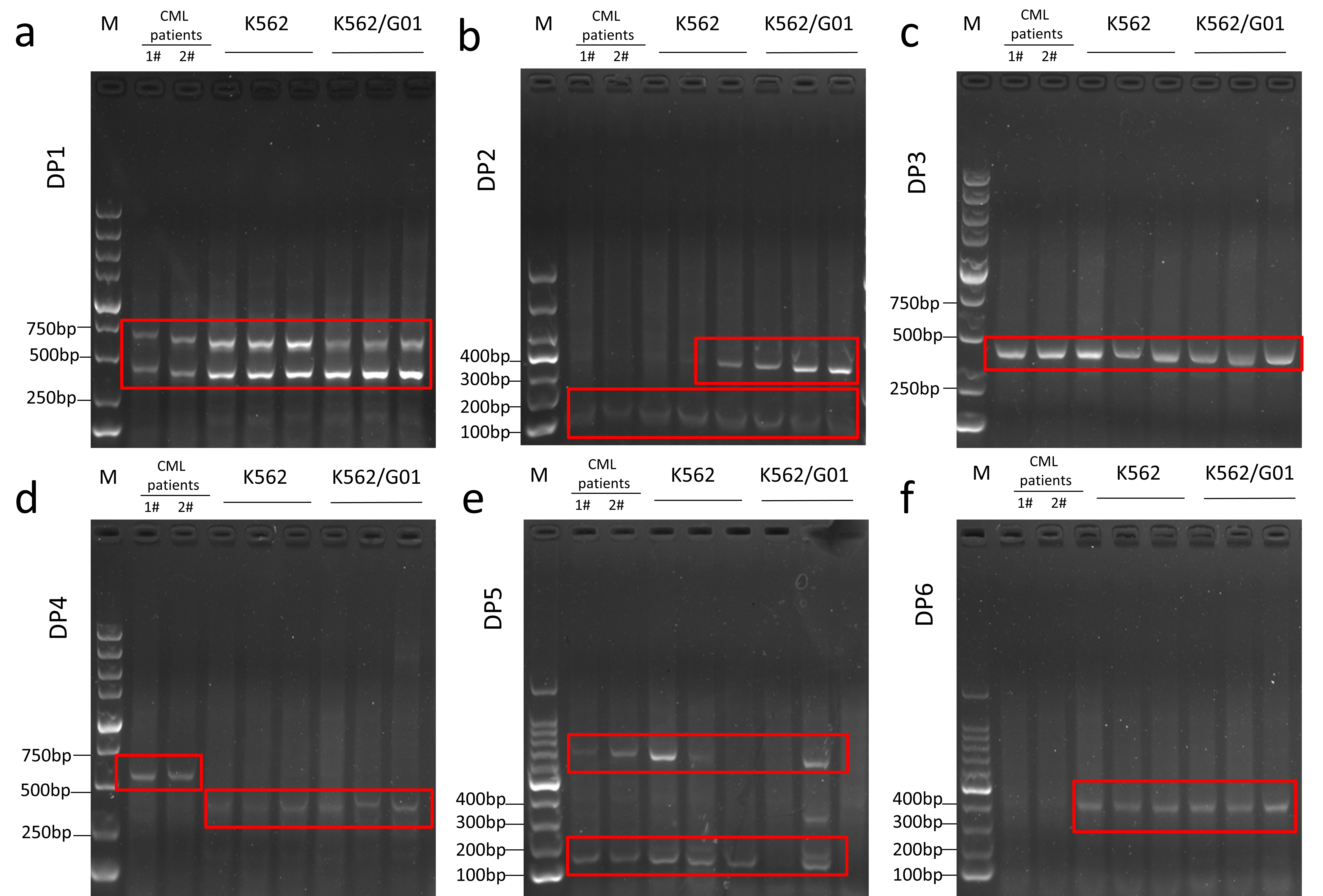

Supplement: Supplemental Material [file KBIE_A_1957749_SM4560.zip › supplementary/Supplementary figure 1.tif]

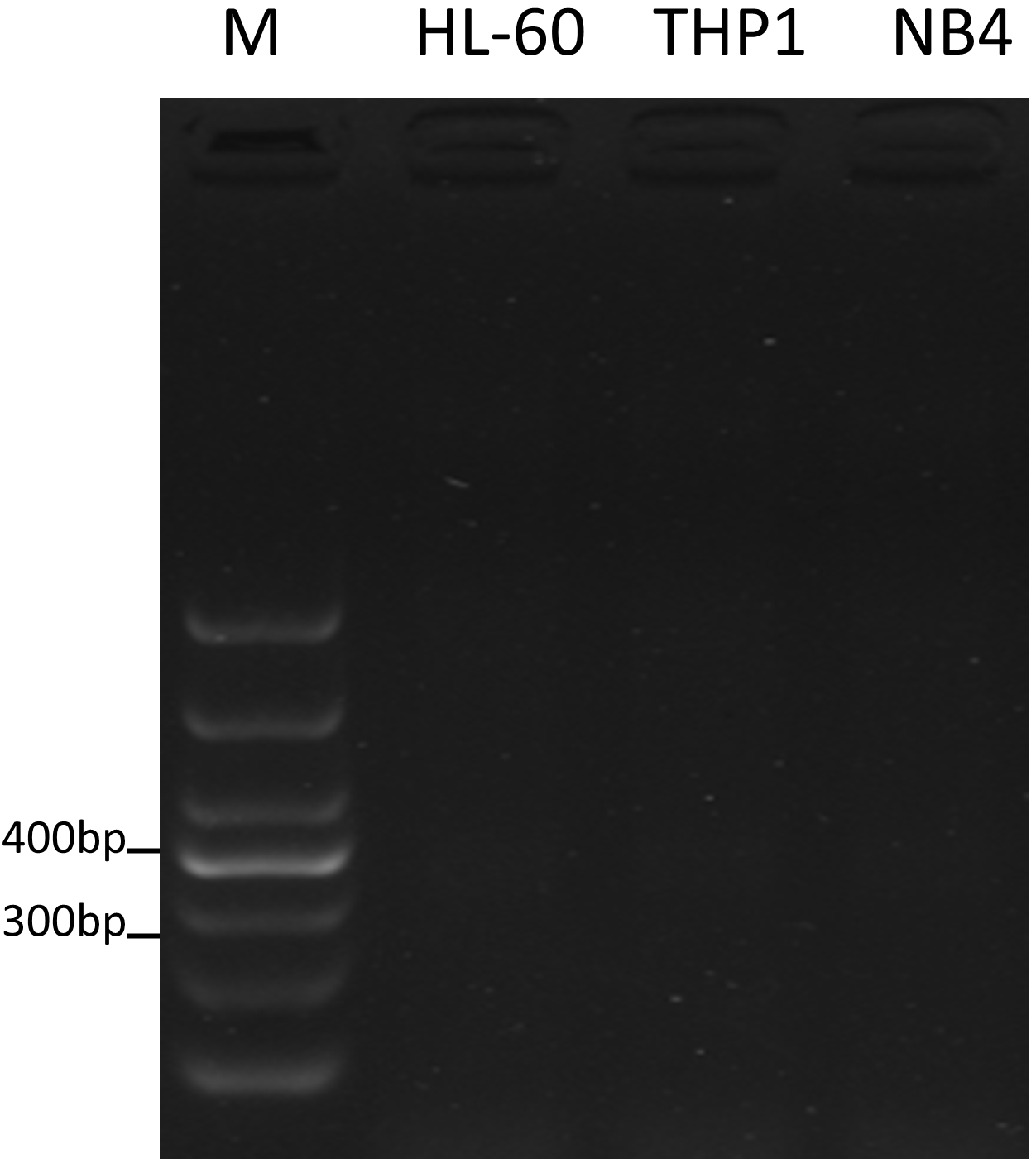

Supplement: Supplemental Material [file KBIE_A_1957749_SM4560.zip › supplementary/Supplementary figure 2.tif]

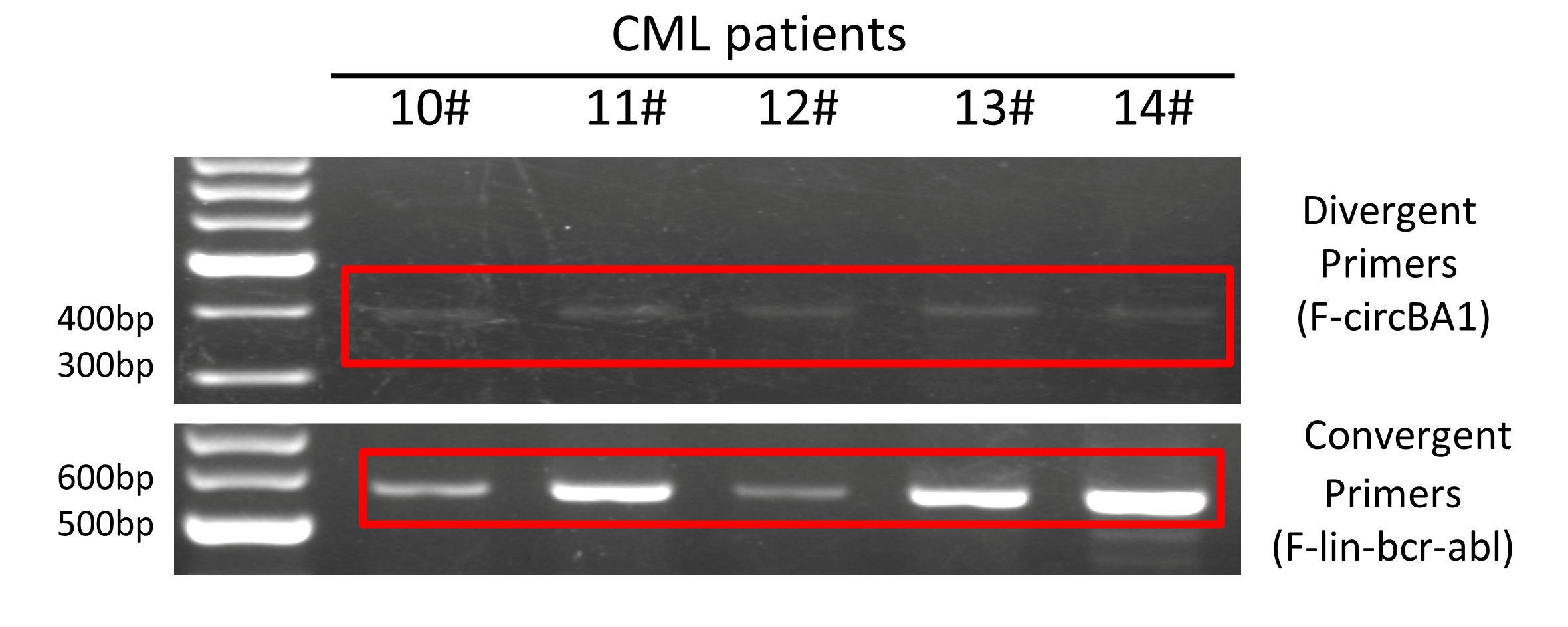

Supplement: Supplemental Material [file KBIE_A_1957749_SM4560.zip › supplementary/Supplementary figure 3.tif]

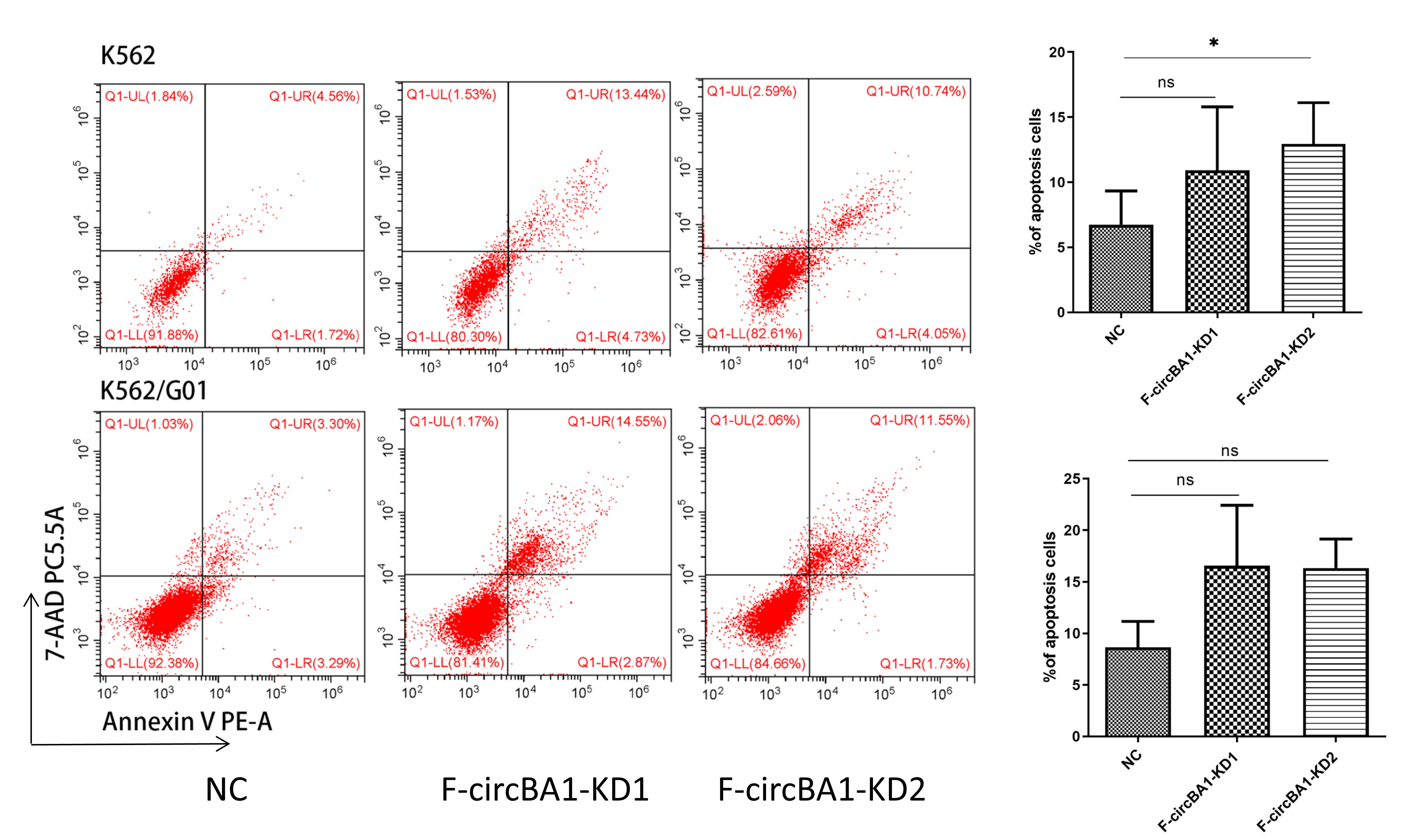

Supplement: Supplemental Material [file KBIE_A_1957749_SM4560.zip › supplementary/Supplementary figure 4.tif]

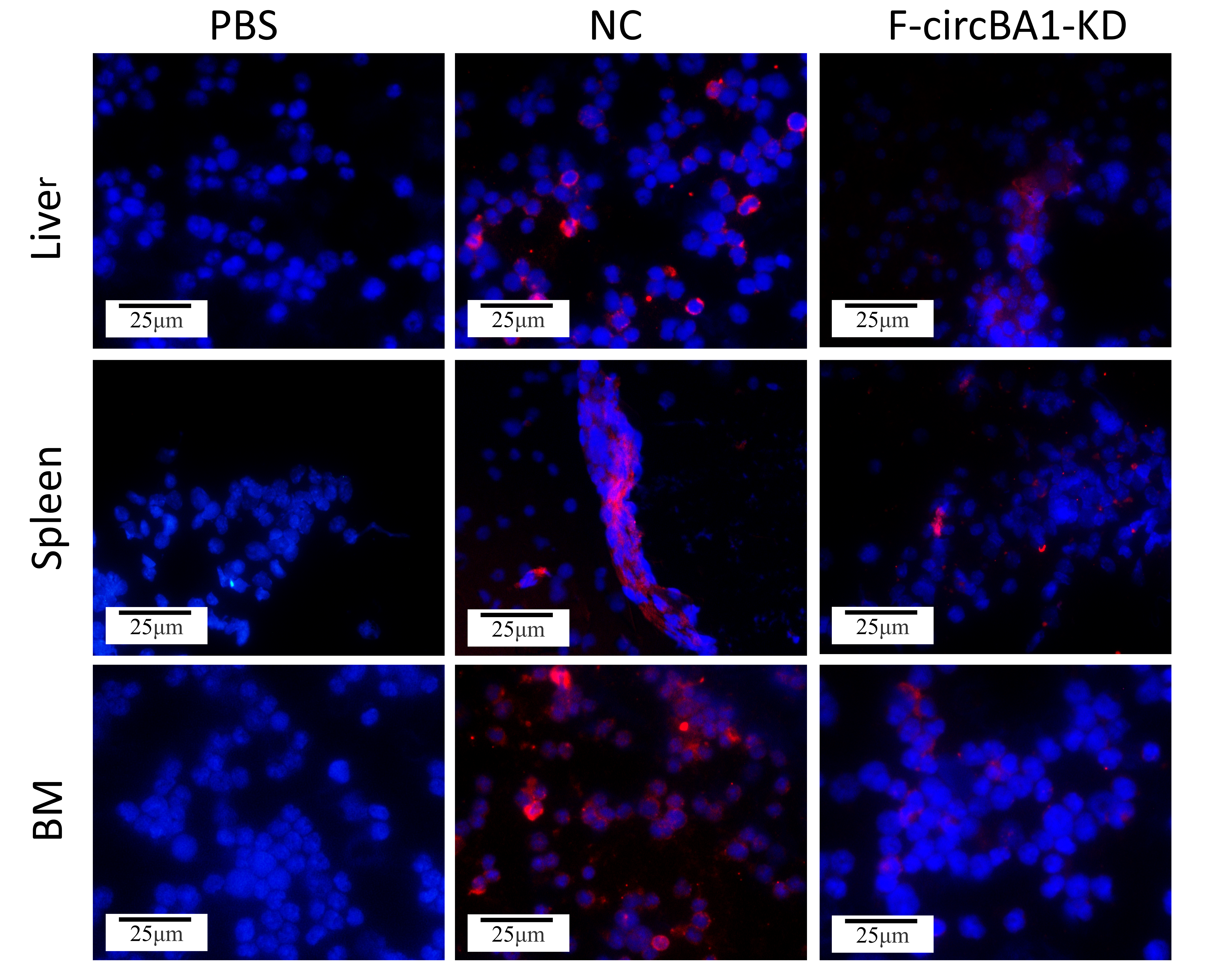

Supplement: Supplemental Material [file KBIE_A_1957749_SM4560.zip › supplementary/Supplementary figure 5.tif]
